# An exploratory survey study of disorder and its association with safety culture in four hospitals

**DOI:** 10.1186/s12913-022-07930-6

**Published:** 2022-04-21

**Authors:** Kate Churruca, Louise A. Ellis, Janet C. Long, Chiara Pomare, Winston Liauw, Caroline M. O’Donnell, Jeffrey Braithwaite

**Affiliations:** 1 Australian Institute of Health Innovation, Macquarie University, Level 6, 75 Talavera Rd, North Ryde, NSW 2113 Australia; 2 St George Hospital, Kogarah, NSW Australia; 3 University of New South Wales, Kensington, NSW Australia; 4 Blacktown and Mt Druitt Hospital, Blacktown, NSW Australia

**Keywords:** Safety culture, Hospital, Physical disorder, Social disorder, Patient safety, Broken windows theory

## Abstract

**Background:**

Signs of disorder in neighbourhoods (e.g., litter, graffiti) are thought to influence the behaviour of residents, potentially leading to violations of rules and petty criminal behaviour. Recently, these premises have been applied to the hospital context, with physical and social disorder found to have a negative association with patient safety. Building on these results, the present study investigates whether physical and social disorder differ between hospitals, and their relationship to safety culture.

**Methods:**

We conducted a cross sectional survey with Likert-style and open response questions administered in four Australian hospitals. All staff were invited to participate in the pilot study from May to September 2018. An analysis of variance (ANOVA) was used to examine differences in disorder by hospital, and hierarchical linear regression assessed the relationship of physical and social disorder to key aspects of safety culture (safety climate, teamwork climate). Open responses were analysed using thematic analysis to elaborate on manifestations of hospital disorder.

**Results:**

There were 415 survey respondents. Significant differences were found in perceptions of physical disorder across the four hospitals. There were no significant differences between hospitals in levels of social disorder. Social disorder had a significant negative relationship with safety and teamwork climate, and physical disorder significantly predicted a poorer teamwork climate. We identified five themes relevant to physical disorder and four for social disorder from participants’ open responses; the preponderance of these themes across hospitals supported quantitative results.

**Conclusions:**

Findings indicate that physical and social disorder are important to consider in attempting to holistically understand a hospital’s safety culture. Interventions that target aspects of physical and social disorder in a hospital may hold value in improving safety culture and patient safety.

**Supplementary Information:**

The online version contains supplementary material available at 10.1186/s12913-022-07930-6.

## Introduction

Since at least the 1980s, sociologists, social psychologists and criminologists have been interested in the concept of “disorder”, including how it is perceived and whether, and in what ways, it is related to anti-social behavior or other negative outcomes [1]. This interest is readily apparent in “Broken Windows Theory” (BWT), a model of neighborhood decline that posits a relationship between visible signs of disorder, on the one hand, and petty criminal behaviour, on the other [2]. Disorder is thought to signify a breakdown in social control; the kinds of incivilities or petty criminal behaviours that are tolerated in an area [3, 4]. Testing this premise in field experiments, Keuschnigg and Wolbring [5] showed that signs of disorder (e.g., litter) increase not only the likelihood of more littering, but also other violations of social norms (e.g., not helping others, stealing). The presence of neighborhood disorder has also been associated with poorer health, and anxiety and depression among residents [6].

In BWT research, it is common to mark a distinction between physical and social disorder. The former relates to the overall physical appearance of a neighbourhood, and includes the eponymous broken windows, vandalism, vacant housing, unkempt property, and litter, while social disorder more directly involves people’s behaviour (e.g., harassment, teenage gangs, fights, drug dealing) [4, 7]. Over the years, two dominant approaches to measuring disorder have emerged, focusing either on structured observation by non-participant researchers, or by asking people who inhabit a space their perceptions of disorder using surveys [8]. Researcher observation reflects an “outsiders’” view on disorder, [4] while Hinkle and Yang [8] make the point that from its genesis BWT posited that people’s *perceptions* of disorder influence their behaviour and thereby crime.

Despite sustained interest in disorder, and its potential implications for understanding social behaviour, the concept has only been applied in a limited fashion to defined, less anonymous spaces like schools [9] or offices [10]. We identified trends in health services research that suggest the concept of disorder has relevance to hospitals [11]. These include the tendency for deviations from rules to become normalized [12, 13] and the association between hospital staff’s perceptions of their work area as cluttered and disorderly and their tendency to comply with safe work practices [14]. Based on these suggestions, we proposed that disorder may be an important construct to consider in hospitals, perpetuating a range of behaviours among staff with potential downstream effects on the quality and safety of care delivered to patients [11]. Although the concept of disorder is nascent in health services research, these ideas are now being considered [15]^.^

Recently, we tested the validity of the disorder construct in hospitals [16]. From a survey of staff across four hospitals, we identified a significant association between physical and social disorder and burnout, job satisfaction and perceptions of patient safety. While this highlights the promise in measuring disorder to “check the temperature” on a range of other important outcomes, the potential for disorder to act as a red flag is dependent on it showing some degree of variance between hospitals.

We also need to understand how perceptions of social and physical disorder may influence safety in hospitals. Safety culture, comprising the shared values, norms and behaviours of staff in relation to patient safety, is a potential candidate in this regard. A link between collective perceptions of disorder and subsequent norms and behaviour related to rule-breaking and incivility is well-established within BWT [5]. The present study extends upon this point, and our other work on BWT, [11, 16] using additional data from the survey of four hospitals to examine whether perceptions of social and physical disorder impact staff’s values, norms and behaviours specifically related to patient safety. Supporting this possibility, one of the earliest surveys to assess safety culture in healthcare included the cleanliness and orderliness of the work environment as a dimension, [14] however this tool has largely been supplanted by other surveys [17]. Hence, while there is good evidence to suggest a relationship between safety culture and perceptions of disorder, much of this work is now decades old, does not assess both physical and social disorder, and does not look at disorder on a hospital-level.

We sought, therefore, to understand the features of physical and social disorder in hospitals and their association with safety culture. Accordingly, this study aimed to explore the concept of disorder in four hospitals through the following research questions:

1. Are there differences between hospitals in the levels and manifestation of physical and social disorder, and if so, what are they?
2. Do perceptions of physical and social disorder significantly predict safety culture in hospitals?

### Hypotheses

For research question 2, the following hypotheses were formulated:

- H1. Physical and social disorder will have significant negative relationships with safety climate, the most central dimension of safety culture.
- H2. Physical and social disorder will have significant negative relationships with teamwork climate, another key dimension of safety culture.

We based our hypotheses on accounts of BWT that focus on spreading norm violations, [3] where signs of the violation of social norms (e.g., litter near a “NO LITTER” sign) are thought to lead to further violations of the same and other norms [5]. Here norms include behaviours related to patient safety, professional conduct and teamwork, which can be classifed as injunctive norms within a hospital and part of its safety culture [3].

## Methods

This was a multi-site survey study designed to pilot test associations between safety culture and disorder variables, and further explore their relationships using quantitative and qualitative data. We adopted a post-positivist paradigm for this analysis, one which assumes an objective social reality in which unobservable phenomena exist that explain the functions of observable events; our comprehension of these explanations is, however, always a subjective approximation [18, 19]. The ethical conduct of this study was approved by South Eastern Sydney Local Health District (HREC ref no: 16/363). All participants implied their consent to participate by completing the survey after reading its front information page. Governance approvals to conduct the research were obtained for each site.

### Sites

Study sites were four major (> 200 beds) public hospitals in metropolitan Australia selected based on broad similarity in the types of services offered (e.g., emergency department, intensive care, surgical, medical, aged care). The four hospitals are part of separate Local Health Districts in New South Wales.

### Participants and recruitment

All clinical and non-clinical staff working at each of the four hospitals were invited to take part in the survey study. Recruitment invitations for the survey were sent to hospital staff via email distribution lists by partner investigators and flyers were also placed around the hospitals. Ethics approval and a lack of funding for this pilot study precluded more active recruitment strategies (e.g., promoting the survey directly on hospital wards), however, a target minimum of 100 respondents per hospital was set based on planned statistical analyses and in accordance with ethics approval from the South Eastern Sydney Local Health District’s Human Research Ethics Committee. In recruitment materials, the study title was deliberately broad, and the description focused on “how staff perceive their workplace, including the physical orderliness of workspaces and the social environment”. We thought this neutral language would be more inclusive of a potentially wider range of views and avoid overly-prescribing what the concept of social disorder should look like in a hospital given the study’s exploratory aims.

### Data collection

The survey commenced with basic demographic questions, followed by quantitative closed response Likert-style questions asking about perceptions of hospital social and physical disorder, and safety culture (strongly disagree = 1 to strongly agree = 5). The development of subscales measuring perceptions of physical and social disorder is described in greater detail in the validation study [16]. In brief, three items comprised the subscale for physical disorder, two were modified from existing measures of physical disorder in neighbourhoods and schools [9, 20] and one we developed based on literature review. Three items made up the subscale for social disorder and were adapted from a study of dishonest workplace behaviours by Coyne and Bartram [21]; this measure was chosen because it was not about healthcare specific (i.e., patient safety) violations, avoiding overlap with safety culture items. All items underwent a round of content validation by a sample (*n* = 10) of nurses, doctors, hospital managers and health services researchers. The internal consistency reliability for both subscales was good (Cronbach’s alpha = 0.84 for physical disorder and 0.86 for social disorder) and, in the previous validation study, confirmatory factor analysis supported their structural validity [16].

We assessed safety culture using two subscales from the Safety Attitude Questionnaire (SAQ): the teamwork climate and safety climate subscales, each with six items [22]. These subscales are frequently used together in lieu of the full SAQ to reduce survey length and because they are most commonly associated with patient outcomes [23]. The survey also included qualitative open response questions to collect additional insights into the manifestation of disorder in hospitals. The first asked about perceptions of aspects of physical disorder in the hospital, and the second examined perceptions of aspects of social disorder in the hospital, but was phrased in a more accessible way (i.e., “behaviour of people in this hospital that negatively affect peace, cooperation and well-coordinated work”) [24]. Additional File 1 provides all relevant survey questions. Survey responses were collected from the hospitals between May and September 2018. The survey was completed either on the online survey platform *Qualtrics* or in hardcopy.

### Data analysis

We performed two one-way analyses of variance (ANOVA) to test for differences between the four hospitals in staff’s perceptions of physical and social disorder. Hierarchical linear regressions were then conducted to determine the extent to which physical disorder and social disorder explained variation in both teamwork climate and safety climate. For each regression, we entered hospital into the model at the first block to determine the degree to which the hospital at which the respondent worked explained variance in their perceptions of the hospital culture (teamwork climate, safety climate). In the second block, physical and social disorder were entered simultaneously as predictors to determine the amount of variance in safety climate and teamwork climate they explained above and beyond variability in climate associated with the hospital the respondent worked at. To facilitate use of nominal predictors in the regression, we coded hospitals into dummy dichotomous variables with Hospital 1 acting as a reference group [25]. Quantitative analyses were conducted in SPSS Statistics, Version 25 [26]. The statistical threshold for all analyses was set at *p* < 0.05.

We conducted a thematic analysis on open response questions to further explore manifestations of disorder across the four hospitals. Two authors (KC, CP) collaboratively developed draft coding frameworks for physical and social disorder based on literature [9, 20, 27]. Following familiarization with the data, we added further codes to ensure code categories adequately covered the content and meaning of all the responses. We discussed these categories with the research team then coded all responses to capture as many codes as were present in each response, with cross coding conducted to ensure consistency. Coded responses were aggregated according to conceptually related topics; they were interpreted thematically through analytical narratives,[28] and comparisons between hospitals were made quantitatively by comparing the proportion of responses focused on that theme at each site.

## Results

Four hundred and fifteen people filled in the survey across the four hospitals. Hospitals varied in the number of respondents. Demographic characteristics of respondents are displayed in Table 1. Means and standard deviations for disorder and safety culture variables by hospital are presented in Table 2. A subscale score was not calculated where a participant did not respond to every item, resulting in an average loss of 9.5% of respondents across the scales.

**Table 1:** Characteristics of survey respondents by hospital

	**Hospital 1 n (%)**	**Hospital 2 n (%)**	**Hospital 3 n (%)**	**Hospital 4 n (%)**
***Sex***				
Male	13 (12.6)	29 (24.4)	25 (17.2)	17 (50)
Female	90 (87.4)	90 (75.6)	120 (82.8)	17 (50)
***Years at hospital***				
< 1 year	13 (12.6)	13 (10.8)	15 (10.4)	3 (8.8)
1–2 years	21 (20.4)	14 (11.7)	11 (7.6)	5 (14.7)
3–5 years	23 (22.3)	33 (27.5)	29 (20.1)	4 (11.8)
6–10 years	16 (15.5)	26 (21.7)	39 (27.1)	10 (29.4)
> 11 years	30 (29.1)	34 (28.3)	50 (34.7)	12 (35.3)
***Role***				
Admin/Clerical	27 (26)	14 (11)	23 (16)	2 (6)
Allied health professionals	21 (20)	5 (4)	31 (21)	0
Management	6 (6)	7 (6)	20 (14)	0
Physician/Medical officer	16 (16)	9 (7)	19 (13)	30 (88)
Registered or enrolled Nurse	24 (23)	68 (54)	36 (25)	2 (6)
Other, including volunteers, scientists, pharmacists, maintenance	9 (9)	22 (18)	17 (12)	0
**Total**	104	127	150	34

**Table 2:** Mean and standard deviation for each variable by hospital

**Hospital**	**Physical disorder**^a^**M (SD)**	**Social disorder M (SD)**^a^	**Safety climate M (SD)**^b^	**Teamwork climate M (SD)**^b^
Hospital 1	3.69 (0.81)	2.16 (0.95)	3.60 (0.73)	3.73 (0.73)
Hospital 2	2.47 (1.03)	2.32 (0.98)	3.62 (0.60)	3.72 (0.74)
Hospital 3	2.98 (0.89)	2.28 (0.81)	3.69 (0.62)	3.73 (0.69)
Hospital 4	2.49 (0.71)	2.02 (0.63)	3.54 (0.62)	3.76 (0.45)
**Total**	2.96 (1.02)	2.22 (0.89)	3.63 (0.64)	3.73 (0.70)

^a^ Response range is from 1–5 with higher scores indicating greater levels of perceived social/physical disorder

^b^ Response range is from 1–5 with higher scores indicating stronger or more positive safety/teamwork climate

### Differences between hospitals in perceptions of physical and social disorder

Following data cleaning and the removal of mostly incomplete responses, there were 360 valid responses on the questions related to disorder. Using a one-way ANOVA, we identified a significant difference among the four hospitals in levels of physical disorder (*F*(3, 387) = 36.59, *p* < 0.001). However, there was no significant difference between hospitals in levels of perceived social disorder (*F*(3, 363) = 1.07, *p* = 0.362). Due to the small sample size from Hospital 4 and the assumption of homogeneity of variance not being met, the Games-Howell post hoc comparison was used to examine differences between hospitals. It showed that staff at Hospital 1 perceived significantly higher levels of physical disorder than those working at Hospital 2 (*M*_*Diff*_ = 1.22, *p* < 0.001, 95% CI [0.90–1.54]), Hospital 3 (*M*_*Diff*_ = 0.72, *p* < 0.001, 95% CI [0.43–1.01]), and Hospital 4 (*M*_*Diff*_ = 1.20, *p* < 0.001, 95% CI [0.81–1.59]). Staff working at Hospital 3 also perceived significantly higher levels of physical disorder than those respondents from Hospital 2 (*M*_*Diff*_ = 0.50, *p* < 0.001, 95% CI [0.19–0.81]) and Hospital 4 (*M*_*Diff*_ = 0.48, *p* < 0.001, 95% CI [0.10–0.87]).

### Relationship of physical and social disorder to safety culture

There were significant intercorrelations among disorder and safety culture variables, with correlations evaluated at the individual respondent level (see Table 3). Social and physical disorder were significantly positively correlated with each other, and both were negatively correlated with safety and teamwork climate scores. Safety and teamwork climate had a strong positive correlation.

**Table 3:** Correlation matrix of disorder and safety culture variables

Variables	Social Disorder	Safety Climate	Teamwork Climate
1. Physical Disorder	.141^a^	-.133^b^	-.139^a^
2. Social Disorder	-	-.399^a^	-.352^a^
3. Safety Climate	-	-	.774^a^
4. Teamwork Climate	-	-	-

^a^ Significant at *p* < .01 (2-tailed)

^b^ Significant at *p* < .05 (2-tailed)

#### The relationship of physical and social disorder to safety climate

Using a hierarchical linear regression, we examined whether physical and social disorder predicted safety climate scores above and beyond the variance accounted for by hospital (see Table 4). In the model, Hospitals 2–4 were entered in Block 1, with Hospital 1 acting as the reference group. This accounted for a nonsignificant amount of the variance in safety climate. With the addition of physical and social disorder at Block 2, the model accounted for a significant 17.5% of the variance in safety climate (*p* < 0.001). However, only social disorder was a significant unique predictor of safety climate, with higher levels of social disorder associated with lower levels of safety climate, providing partial support to H1.

**Table 4:** Hierarchical linear regression of safety climate

		**Unstandardized**		**Standardized**	
**Block**	**Variables**	***B***	**SE**	**β**	**Sig**
1—Hospital	*R*^*2*^ = .006, *F* = 0.686 [3, 363]				.561
	Hospital 2^a^	.010	.091	.007	.913
	Hospital 3^a^	.092	.088	.069	.297
	Hospital 4^a^	-.056	.136	-.024	.678
2—Disorder	*R*^*2*^ = .175, Δ*R*^*2*^ = .169, Δ*F* = 36.726 [5, 363]				.000^b^
	Hospital 2^a^	-.021	.093	-.015	.824
	Hospital 3^a^	.064	.084	.047	.452
	Hospital 4^a^	-.166	.130	-.071	.202
	Physical Disorder	-.060	.034	-.095	.084
	Social Disorder	-.281	.035	-.389	.000^b^

^a^Hospital 1 was the reference group

^b^ Significant at *p* < .001

#### The relationship of physical and social disorder to teamwork climate

We ran a second hierarchical linear regression to assess the extent to which physical and social disorder predicted teamwork climate scores once hospital was taken into consideration. Hospitals 2–4 were entered at Block 1, with Hospital 1 once again the reference group (see Table 5 for results). The hospital in which respondents worked at did not significantly predict their perceptions of teamwork climate. When physical and social disorder were added at Block 2, the model accounted for a significant 13.5% of the variance in teamwork climate (*p* < 0.001). Both physical and social disorder significantly contributed to the prediction of teamwork climate; the associations were negative, providing support for H2.

**Table 5:** Hierarchical linear regression of teamwork climate

		**Unstandardized**		**Standardized**	
**Block**	**Variables**	***B***	**SE**	**β**	**Sig**
1—Hospital	*R*^*2*^ = .000, *F* = 0.016 [3, 366]				.997
	Hospital 2^a^	-.007	.098	-.004	.946
	Hospital 3^a^	-.015	.095	-.010	.876
	Hospital 4^a^	.012	.145	.005	.936
2—Disorder	*R*^*2*^ = .135, Δ*R*^*2*^ = .135, Δ*F* = 28.237 [5, 366]				.000^c^
	Hospital 2^a^	-.061	.103	-.040	.554
	Hospital 3^a^	-.056	.093	-.038	.548
	Hospital 4^a^	-.117	.142	-.047	.411
	Physical Disorder	-.080	.038	-.117	.037^b^
	Social Disorder	-.263	.039	-.336	.000^c^

^a^ Hospital 1 was the reference group

^b^ Significant at *p* < .05

^c^ Significant at *p* < .001

### Qualitative results

Around half of survey participants (*n* = 210, 50.6%) provided at least one response to the open ended questions. Themes are discussed below for the responses to physical and social disorder questions.

#### Physical disorder

For the open response question on physical disorder, we identified five themes with a number of subthemes summarized in Table 6. The most common theme across hospitals, and particularly in Hospital 1 and Hospital 3, was *long term and structural issues*. This included *clutter and insufficient space for work*, with staff at Hospital 1 reporting “a lack of equipment storage areas” and workspaces “not fit for purpose”. It was further reflected in *building maintenance* issues*,* such as the mismatch between new and old parts of the same hospital (“new build has left original buildings looking more tired and in need of update”, Hospital 3) and a general sense of poor maintenance: “old building, chaotic layout, lack of maintenance” (Hospital 4), “walls need painting, refurb-very old” (Hospital 3). Finally, a small number of respondents mentioned *aesthetic issues,* like “tatty décor” (Hospital 3).

**Table 6:** Number of times themes and codes related to physical disorder in hospitals were reflected in participant responses

Themes and subthemes	Hospital 1 Count (%)	Hospital 2 Count (%)	Hospital 3 Count (%)	Hospital 4 Count (%)	Total Count (%)
**Long term and structural issues**	26 (55.3)	24 (41.3)	42 (68.8)	5 (55.6)	97 (55.4)
Clutter and insufficient space for work	21 (44.7)	19 (32.7)	35 (57.3)	5 (55.6)	80 (45.7)
Building maintenance	10 (21.3)	7 (12.1)	15 (24.5)	2 (22.2)	34 (19.4)
Aesthetics	1 (2.1)	3 (5.2)	4 (6.6)	0 (0.0)	8 (4.5)
**Human behaviour and day-to-day issues**	25 (53.2)	30 (51.7)	24 (39.3)	2 (22.2)	81 (46.3)
Human behaviour contributes to physical disorder	21 (44.7)	20 (34.5)	19 (31.1)	1 (11.1)	61 (34.9)
Day-to-day upkeep	13 (27.7)	19 (32.8)	13 (21.3)	1 (11.1)	46 (26.2)
Disorganised and unprofessional hospital work practices	9 (19.1)	7 (12.1)	13 (21.3)	1 (11.1)	30 (17.1)
**Resourcing issues**	8 (17.0)	16 (25.6)	6 (9.8)	1 (11.1)	31 (17.7)
Inadequate equipment	3 (6.4)	9 (15.5)	4 (6.6)	0 (0.0)	16 (9.1)
Insufficient time and resources for tidiness	6 (12.7)	7 (12.1)	2 (3.3)	1 (11.1)	16 (9.1)
**Risks to patients**	5 (10.6)	1 (1.7)	2 (3.3)	0 (0.0)	8 (4.5)
**Positive perceptions of the hospital physical environment**	1 (2.1)	2 (3.4)	1 (1.6)	0 (0.0)	4 (2.3)
**Total number of responses**	47	58	61	9	175

% based on proportion of participants answering this question

Respondents, especially at Hospital 1 and Hospital 2, highlighted that staff behaviour contributed to hospital physical disorder (*human behaviour and day-to-day issues*). The notion that *human behaviour contributes to physical disorder*, particularly through poor *day-to-day upkeep* included staff not cleaning up (“lack of garbage collection”, Hospital 1) and a general sense that “no one cares about the physical workspace; there is no sense of ownership when it comes to keeping it tidy” (Hospital 2). Besides cleaning, numerous respondents described *disorganized or unprofessional hospital work practices* (e.g., not storing trolleys or computer-on-wheels appropriately). These could sometimes impact patients: “People sharing office space and having no responsibility to tidy up papers … can include confidential patient information” (Hospital 3).

Particularly in Hospital 2, staff linked these problems to *resourcing issues,* such as *inadequate equipment* and *insufficient time and resources for tidiness:* “Lack of time to tidy after end of clinic day” (Hospital 2). These responses implied tidiness was less of a priority than clinical care, however, some respondents linked physical disorder to potential *risks to patients*: “kitchen areas are not cleaned to a standard that would prevent transferring bacteria/infections” (Hospital 1), “Staff leaving mess and expecting it to be "picked up" by others … can border on an infection control risk at times” (Hospital 2). As the final theme, a very small number of participants also reported *positive perceptions of the hospital physical environment,* that “all areas are clean and tidy” (Hospital 3).

#### Social disorder

We identified four broad themes from the 166 responses to the question tapping social disorder; these are summarized in Table 7. A small proportion of participants described *positive perceptions of the social environment* at their hospital, that staff were “very caring of their patients, very polite” (Hospital 2) and there was a “good culture” (Hospital 1). On the negative side, numerous aspects of the *organisational culture* were cited as issues across the four hospitals, such as *self-interest and poor accountability* in which respondents described that irresponsible or “selfish” staff behaviour was not corrected and there was little incentive for good behaviour. *Leadership issues* were also mentioned: “too many people fail to take a bit of extra effort to complete a task … managers do not seem to be concentrating on their staff, but are ensuring that surveys are completed (which can be measured) versus actually delivering nursing care” (Hospital 2). Culture issues also encompassed *poor collaboration,* such as a “general lack of communication, overall lack of collaboration and openness” (Hospital 3), and, less frequently, *resistance to change* and a *punitive environment with the inability to speak up*. *Not following hospital rules and values*, both by consumers (“relatives who will not comply with the 2 visitors at a time policy which creates noise, and overcrowding affecting other patient in the [shared] room”) and staff (“There has been stealing of food from the communal fridge”) was more common in the answers from Hospital 1.

**Table 7:** Number of times themes and subthemes related to social disorder in hospitals were reflected in participant responses

Themes and subthemes	Hospital 1 Count (%)	Hospital 2 Count (%)	Hospital 3 Count (%)	Hospital 4 Count (%)	Total Count %
**Organisational culture issues**	26 (61.9)	26 (52.1)	37 (61.7)	10 (71.4)	99 (59.6)
Self-interest and poor accountability	7 (16.7)	12 (24.0)	15 (27.3)	4 (28.6)	38 (22.9)
Leadership issues	8 (19.0)	10 (20.0)	12 (20.0)	4 (28.6)	34 (20.5)
Poor collaboration	11 (26.9)	5 (10.0)	10 (16.7)	7 (50.0)	33 (19.9)
Not following hospital rules and values	9 (24.4)	4 (8.0)	4 (6.7)	2 (14.3)	19 (11.4)
Resistance to change	0 (0.0)	3 (6.0)	5 (8.3)	2 (14.3)	10 (6.0)
Punitive environment with the inability to speak up	4 (9.5)	1 (2.0)	1 (1.7)	2 (14.3)	8 (4.8)
**Incivility, disrespect and abuse**	18 (42.9)	19 (38.0)	13 (21.7)	3 (21.4)	53 (31.9)
**Challenges in hospital work**	7 (16.7)	13 (26.0)	12 (20.0)	4 (28.6)	36 (21.7)
Workspace and layout issues including clutter and noise	2 (4.7)	2 (4.0)	4 (6.7)	0 (0.0)	8 (4.8)
Problematic work processes and tools	2 (4.7)	1 (2.0)	2 (3.3)	1 (7.1)	6 (3.6)
Workload, understaffing and other constraints	2 (4.7)	6 (12.0)	7 (11.7)	3 (21.4)	18 (10.8)
Patient/Relative involvement	3 (7.1)	4 (8.0)	1 (1.7)	0 (0.0)	8 (4.8)
**Positive perceptions of the social environment**	2 (4.7)	2 (4.0)	2 (3.3)	0 (0.0)	6 (3.6)
**Total number of responses**	42	50	60	14	166

% based on proportion of participants answering this question for each hospital and overall

*Incivility, disrespect and abuse,* including *“*anti-social behaviour, manipulative, self-centred behaviour” (Hospital 1) and the “obstructive nature of other nurses/staff” (Hospital 2), were touched upon by approximately a third of participants. A higher proportion of these responses were from hospitals 1 and 2. Respondents also mentioned a range of *challenges of working in a hospital,* such as *workload, understaffing and other constraints*: “no education provided to workers” (Hospital 4), “unusable equipment” (Hospital 4), “staff shortages” (Hospital 1). *Workspace and layout issues* such as *noise* and lack of privacy common in an “open-plan work area” (Hospital 1) were also reported. The specific challenges of working with patients and relatives ran across numerous responses (*patient/relative involvement)*. Lastly, a few participants at each of the hospitals reported *problematic work processes and tools*, for example, “Too much emphasis on getting things done for hospital inspections (e.g., for hospital accreditation)” (Hospital 1), or the inappropriate “focus on time based KPIs [key performance indicators]” (Hospital 4).

## Discussion

Following interest in using BWT to explain the influence of the environment on human behaviour in healthcare organizations, our study explored how disorder manifests across hospitals and its association with safety culture. Using a validated measure of hospital disorder, we found significant differences in perceived physical disorder between hospitals, but no significant differences in levels of social disorder.

Qualitative findings also suggested variability between hospitals in the degree and types of disorder present; these results elaborated further on manifestations of disorder, indicating the potential for physical disorder to affect patient care through poor infection control or risks to confidentiality, and demonstrating the impact interpersonal issues like incivility and poor accountability have on hospital staff’s capacity to work effectively. Indeed, many of the additional features of physical disorder reported in open responses—neglect of building maintenance, poor day-to-day upkeep and clutter—have been recognized as potential hazards to hospital quality and safety in the research literature [29]. Additional features of social disorder highlighted by our qualitative analysis, such as incivility and abuse, have been found to negatively affect patient care, [30–33] and suggest a different way in which social disorder could be operationalised in hospitals. In this vein, in 2003, Hesketh, Duncan [34] advanced BWT as an explanation for violence among hospital staff and patients.

From the multiple regression analyses, we found that social disorder significantly predicted a hospital’s safety climate, and both physical and social disorder were significant predictors of teamwork climate. In all instances, higher levels of disorder were associated with lower levels of safety or teamwork climate. The significant relationship between social disorder and both teamwork and safety climate aspects of safety culture highlights the negative effect that staff rule breaking has on the prioritisation of patient safety and capacity to work together within hospitals. This extends research finding a relationship between safety culture and compliance with, and participation in, patient safety behaviour, [35] highlighting that safety culture is related to perceptions of whether other staff comply with general workplace behaviour (e.g., late to shift).

In our study, physical disorder predicted teamwork, but not safety climate. Perhaps the reason for this is that the items forming the physical disorder subscale do not as directly address violation of norms as the social disorder items; they arguably more highlight hospital neglect. The relationship between physical disorder and teamwork climate is supported by literature on environmental design, which demonstrates how the layout, design and other physical features of the hospital impact the capacity for communication and collaboration [36].

### Implications

Our research underscores the value of considering disorder when seeking to understand safety and quality in hospitals. Physical disorder, while not directly related to safety climate, may affect teamwork and thereby patient safety. It also showed a greater, more consistent degree of variance across hospitals in our study than social disorder. While it is not easy to refurbish a hospital—this comes with its own host of challenges [37]—the physical environment is more amenable to direct intervention than an organisation’s culture [38].

Our findings indicate that social disorder influences safety and teamwork climate, both of which are important determinants of the safety and quality of care provided to patients in hospitals. However, we should be wary of taking the kind of “zero-tolerance” approach to hospital staff “slacking off” or “disregarding rules” as was applied to petty crimes in neighbourhoods of New York City during the 1990s [39]. Evidence is not persuasive that such strategies are effective, [40] and they may result in the unfair targeting of groups with less power (e.g., cleaners, junior nurses), as they did in neighbourhoods.

Research on BWT in neighbourhoods has in more recent times utilised an experimental study design to examine the social processes underpinning the spread of disorder [3, 41]. However, any such intervention is probably not appropriate in the busy, pressurised and high-stakes environment of hospitals. Another starting point might be a quality improvement project that aims to create a more orderly and organised workplace, and encourages staff to contribute to this. This could include a range of implementation strategies such as engaging leadership, and utilising champions to model best practice in following rules, negotiating when to take breaks, and time management (i.e., aspects of social order) [42]. Indeed, respected senior figures have been shown to have an important role in perpetuating norms in healthcare, such as those related to hand hygiene [43]. This intervention could be evaluated for impacts on group cohesion, teamwork, safety culture and patient safety, as well as on specific behavioural norms.

Conceptually, despite the open response questions separately probing aspects of physical and social disorders, respondents did not readily differentiate between the two; they linked physical disorder to human behaviour, and highlighted workspace issues as affecting staff actions. Similar to the application of BWT in neighbourhoods,[4, 7] there is a degree of overlap between physical and social disorder in hospital [16]. Furthermore, staff explained some instances of disorder as due to limited time and being understaffed, which implied day-to-day upkeep (e.g., cleaning, putting equipment away) was traded off in favor of provision of services to patients [44]. In this sense, disorder may be an indicator of broader issues related to the resourcing of hospitals rather than an issue in itself, which mirrors one perspective on neighborhood disorder that links it and crime to structural constraints such as economic inequality [45].

### Strengths and limitations

The utilisation of qualitative analyses to further explore, corroborate and extend upon quantitiative findings was a strength of this study, one that has expanded our understanding of the potential impacts of disorder on hospital work. We also used validated scales to assess the constructs of disorder [16] and safety culture [22]. A limitation was the limited sample from each hospital and the comparably low response for one; this did not preclude finding significant associations among variables to support most of our hypotheses. Drawing from our findings, larger scale research may be designed to determine the generalisability of relationships among disorder and safety culture constructs. Any future survey research might use more active approaches to recruitment, rather than reliance upon passive strategies (i.e., emails and flyers), which were not as able to capture responses from time poor clinical staff working on wards and away from computers. Missing data was also an issue in the calculation of subscales because in accordance with the ethical principle of respect, we did not compel participants to respond to every question. While our analysis relied exclusively upon self-reported data, we do not see this as a limitation; literature indicates that individuals’ perceptions of disorder are more important in understanding the link between disorder and behaviour, than, for example, researcher observations [8].

## Conclusion

This study explored manifestations of disorder in hospitals, and the association of physical and social disorder with aspects of hospital safety culture. Through a survey of staff across four hospitals, we identified significant differences between hospitals in staff’s perceptions of physical disorder that were corroborated by themes in qualitative open responses. We also found a significant association between social disorder and safety climate, and between physical and social disorder and teamwork climate. These results provide further evidence to support the premise that disorder is important to consider in attempting to understand and improve quality and safety in hospitals.

## Supplementary Information

**Additional file 1.**

## Data Availability

The data analysed during the current study are not publicly available due to ethical restrictions, but are available from the corresponding author on reasonable request and with appropriate ethics approval.

## Abbreviations

BWT: Broken windows theory
CI: Confidence interval
SAQ: Safety Attitudes Questionnaire
