# Supplementary material for: An exploratory survey study of disorder and its association with safety culture in four hospitals
Source: BMC Health Serv Res. 2022 Apr 21;22:530. doi: 10.1186/s12913-022-07930-6 (PMC9026660; doi:10.1186/s12913-022-07930-6)
Supplement: Supplementary file 1 — Additional file 1. [file 12913_2022_7930_MOESM1_ESM.docx]

# Appendix 1

**Survey questions**

| **Area/area** | **Question lead in** | **Question text** | **Answer options/format** |
| --- | --- | --- | --- |
| Demographics | About you | What is your main role/job in this hospital? | - Administration/Clerical - Allied Health Professional - Maintenance, Catering, and Cleaning - Management - Pharmacist - Physician / Medical Officer - Registered Nurse - Scientist/Laboratory Staff - Volunteer - Other (please specify) _______________ |
|  |  | What is your gender? | - Male - Female - Other |
|  |  | What is your age? | - 18-24 years - 25-34 years - 35-44 years - 45-54 years - 55-64 years - 65+ years |
|  |  | How long have you worked at this hospital? | - 3 months or less - 4-6 months - 7-12 months - 1-2 years - 3-5 years - 6-10 years - More than 11 |
|  |  | What is the highest level of education you have attained? | - Completed Year 11 of High School or below - Completed Year 12 of High School - Certificate or diploma (e.g. TAFE, trade qualification) - Undergraduate Degree (e.g. Bachelor's degree) - Graduate Diploma/Graduate Certificate - Postgraduate Degree (e.g. Master's, PhD) |
|  |  | On average, how many hours per week do you work at this hospital? | - Less than 20 hours per week - 20 to 39 hours per week - 40 hours per week or more |
|  |  | What is your primary work area/unit at this hospital? (select one) | - AMI/Cardiology - Emergency Department - Intensive Care Unit - Maternity/Neonatal - Hip Fracture/Orthopedics - Pathology - Pediatrics - Pharmacy - Psychiatric/Mental Health - Radiology - Rehabilitation - Stroke/Neurology - Theatre/Recovery - Other Clinical Area/Ward (please specify) - Administrative Services and Departments - Catering and Food Services - Housekeeping - Medical Records - Other Non-Clinical Area/Department (please specify) |
|  |  | Do you have a formal management role in this hospital? | - Yes - No |
|  |  | If you have a formal management role, which of the following best describes your formal management role? | - Team Leader - Manager - Director of a Service - Executive Staff - N/A |
|  |  | In your job at this hospital, do you have direct interaction or contact with patients? | - Yes - No |
| Disorder/physical disorder | Please indicate your agreement or disagreement with the following statements about this hospital.  Thinking about this hospital ... | This hospital is run-down and in disrepair | - - - 1. Strongly disagree       2. Disagree       3. Neutral       4. Agree       5. Strongly agree |
|  |  | There is a lot of broken equipment in this hospital | - - - 1. Strongly disagree       2. Disagree       3. Neutral       4. Agree       5. Strongly agree |
|  |  | This hospital is in need of refurbishment | - - - 1. Strongly disagree       2. Disagree       3. Neutral       4. Agree       5. Strongly agree |
| Disorder/social disorder | Please indicate to what extent you agree that the following behaviours are a problem in the main area or unit where you work. | Taking breaks without permission | - - - 1. Strongly disagree       2. Disagree       3. Neutral       4. Agree       5. Strongly agree |
|  |  | Disregard for hospital rules, policies and procedures | - - - 1. Strongly disagree       2. Disagree       3. Neutral       4. Agree       5. Strongly agree |
|  |  | Wasting time/"slacking" off at work | - - - 1. Strongly disagree       2. Disagree       3. Neutral       4. Agree       5. Strongly agree |
| Safety culture/safety climate | Please indicate your agreement or disagreement with the following statements | I would feel safe being treated here as a patient | - - - 1. Strongly disagree       2. Disagree       3. Neutral       4. Agree       5. Strongly agree |
|  |  | Medical errors are handled appropriately in this unit | - - - 1. Strongly disagree       2. Disagree       3. Neutral       4. Agree       5. Strongly agree |
|  |  | I receive appropriate feedback about my performance | - - - 1. Strongly disagree       2. Disagree       3. Neutral       4. Agree       5. Strongly agree |
|  |  | In my work area, it is difficult to discuss errors | - - - 1. Strongly disagree       2. Disagree       3. Neutral       4. Agree       5. Strongly agree |
|  |  | I am encouraged by my colleagues to report any patient safety concerns I may have | - - - 1. Strongly disagree       2. Disagree       3. Neutral       4. Agree       5. Strongly agree |
|  |  | The culture in my work area makes it easy to learn from the errors of others | - - - 1. Strongly disagree       2. Disagree       3. Neutral       4. Agree       5. Strongly agree |
| Safety culture/teamwork climate | Please indicate your agreement or disagreement with the following statements | Nurse input is well received in my work area | - - - 1. Strongly disagree       2. Disagree       3. Neutral       4. Agree       5. Strongly agree |
|  |  | In my work area, it is difficult to speak up if I perceive a problem with patient care | - - - 1. Strongly disagree       2. Disagree       3. Neutral       4. Agree       5. Strongly agree |
|  |  | Disagreements in my work area are resolved appropriately (i.e. not who is right, but what is best for the patient) | - - - 1. Strongly disagree       2. Disagree       3. Neutral       4. Agree       5. Strongly agree |
|  |  | I have the support I need from other staff in my unit to care for patients | - - - 1. Strongly disagree       2. Disagree       3. Neutral       4. Agree       5. Strongly agree |
|  |  | It is easy for staff in this unit to ask questions when there is something that they do not understand | - - - 1. Strongly disagree       2. Disagree       3. Neutral       4. Agree       5. Strongly agree |
|  |  | The doctors and nurses here work together as a well-coordinated team | - - - 1. Strongly disagree       2. Disagree       3. Neutral       4. Agree       5. Strongly agree |
| Disorder/physical disorder | Final Questions and Comments | Are there other things you notice about your work area that contribute to it looking messy, untidy or disorderly? (Please list them) | ________________________________  (Open response - four lines for hard copy, unrestricted number of characters in a box for online survey) |
| Disorder/social disorder |  | Are there other things you notice about the behavior of people in this hospital that negatively affect peace, cooperation and well-coordinated work? (Please list them) | ________________________________  (Open response - four lines for hard copy, unrestricted number of characters in a box for online survey) |
